# Cytotoxicity of Superoxide Dismutase 1 in Cultured Cells Is Linked to Zn^2+^ Chelation

**DOI:** 10.1371/journal.pone.0036104

**Published:** 2012-04-25

**Authors:** Ann-Sofi Johansson, Monika Vestling, Per Zetterström, Lisa Lang, Lina Leinartaitė, Mikael Karlström, Jens Danielsson, Stefan L. Marklund, Mikael Oliveberg

**Affiliations:** 1 Department of Biochemistry and Biophysics, Stockholm University, Stockholm, Sweden; 2 Department of Medical Biochemistry and Biophysics, Umeå University, Umeå, Sweden; 3 Department of Medical Biosciences, Clinical chemistry, Umeå University, Umeå, Sweden; Mental Health Research Institute of Victoria, Australia

## Abstract

Neurodegeneration in protein-misfolding disease is generally assigned to toxic function of small, soluble protein aggregates. Largely, these assignments are based on observations of cultured neural cells where the suspect protein material is titrated directly into the growth medium. In the present study, we use this approach to shed light on the cytotoxic action of the metalloenzyme Cu/Zn superoxide dismutase 1 (SOD1), associated with misfolding and aggregation in amyotrophic lateral sclerosis (ALS). The results show, somewhat unexpectedly, that the toxic species of SOD1 in this type of experimental setting is not an aggregate, as typically observed for proteins implicated in other neuro-degenerative diseases, but the folded and fully soluble apo protein. Moreover, we demonstrate that the toxic action of apoSOD1 relies on the protein's ability to chelate Zn^2+^ ions from the growth medium. The decreased cell viability that accompanies this extraction is presumably based on disturbed Zn^2+^ homeostasis. Consistently, mutations that cause global unfolding of the apoSOD1 molecule or otherwise reduce its Zn^2+^ affinity abolish completely the cytotoxic response. So does the addition of surplus Zn^2+^. Taken together, these observations point at a case where the toxic response of cultured cells might not be related to human pathology but stems from the intrinsic limitations of a simplified cell model. There are several ways proteins can kill cultured neural cells but all of these need not to be relevant for neurodegenerative disease.

## Introduction

In neurodegenerative diseases, the pathogenesis has in multiple cases been linked to misfolding and aggregation of proteins and peptides [1]. The mechanism by which these misfolded or aggregated proteins exert toxicity to neural cells, however, is not clear. One reason is that studies of misfolding and aggregation phenomena are generally complicated by the elusive nature of unstructured and partly unfolded proteins. Also, the actual toxicity response is often difficult to pin down at physiological level due to the complexity of even the most simplistic cell models. To shed further light on these issues we examine here the cytotoxic response of cultured neuroblastoma cells to the metal-coordinating enzyme Cu/Zn superoxide dismutase (SOD1) implicated in the neurodegenerative disorder amyotrophic lateral sclerosis (ALS). A particular advantage of this model is that SOD1 has a well-characterized three-dimensional structure [2] that is amenable to a wide spectrum of biophysical analyses as well as extensive modifications by protein engineering [3], [4], [5]. In addition, by using a fairly simple cell model where the protein is added directly to the cell media, the concentration and biophysical properties of SOD1 can be more accurately controlled. Disease relevance of this reductionist model is provided by the implicated extracellular role of SOD1 in propagating damage in the central nervous tissue. Even though SOD1 exist as an intracellular protein *in vivo*, and hence ALS is most likely triggered intracellularly, the neural damage seems to be able to propagate to neighbouring cells [6]. As an explanation to how this occurs, SOD1 has been demonstrated to interact with chromogranins, a component of secretory vesicles, possibly promoting the secretion of SOD1 [7]. The contribution of extracellular SOD1 is further highlighted by the ability of SOD1 to trigger neural death when added extracellularly to cultured cells [7] as well as in stem-cell derived motor neurons cultured together with glia cells expressing mutant SOD1 [8], [9].

The SOD1 gene comprises >140 different missense mutations associated with familial ALS [10], which provide a uniquely large reference set for mapping out the molecular determinants of the disease. Accordingly, the most likely precursor for ALS has been identified as the fully, or partly, metal-depleted apoSOD1 protein [3], [11], [12], and the mutational perturbations that seem to inflict disease are decreased protein stability [3], [11], [13] and reduced repulsive charge [14]. Even so, the data relating to how apoSOD1 induces neural damage have been quite disparate, illustrating partly the complexity of inferring disease mechanism from simplistic cellular model systems. One line of evidence favours toxic aggregation [15], [16], [17] and overload of the cellular housekeeping system [18], [19], whereas another points at noxious radical chemistry catalysed by the Zn^2+^-depleted enzyme [20]. By designing the experiment so that neither of these putative toxic pathways can be engaged, we demonstrate here that SOD1 is still able to exert toxicity to cultured cells. The basis for this toxicity, as it turns out, involves an intact un-metallated Zn^2+^-site, as addition of Zn^2+^ to the culture media or disruption of the Zn^2+^-site using protein engineering completely inhibits toxicity. Thus, the toxic response of apoSOD1 closely resembles the toxicity of low molecular weight Zn^2+^ chelators, i.e. apoSOD1 presumably disturbs cellular Zn^2+^ homeostasis, thereby causing a cytotoxic response. This additional toxicity effect of apoSOD1 underlines not only the multiplicity of ways cultured cells can respond to a single disease-associated protein, but is also interesting by exposing a case where the physiological relevance of the cellular response is questionable. The toxic action of apoSOD1 in cell cultures is unlikely part of the human ALS mechanism.

## Results

### Monomeric and dimeric apoSOD1 reduce cell viability of cultured neuroblastoma cells

The active SOD1 enzyme exists within the cell as a dimeric metallated holo protein, with each subunit containing one redox-active Cu^1+/2+^ ion and one Zn^2+^ ion. However, a small fraction of the SOD1 molecules are expected to reside in their metal-free apo form, as metal loading is dependent on the availability of metals as well as the copper chaperone for SOD1 (CCS) [21]. To be able to study the monomeric protein, two dimer splitting mutations were introduced; F50E and G51E [22]. In addition, in both monomeric and dimeric protein, C6 and C111 were substituted for alanine to avoid aggregation by disulfide cross-linking [23], [24]. Notably, this mutant leaves the intramolecular disulphide bridge between C57 and C146 intact. The monomeric [C6/111A;F50E;G51E] protein is throughout the paper referred to as monomer, and the [C6/111A] protein as dimer. To assess the impact on cell viability of these proteins, monomeric and dimeric SOD1 in apo or holo form were added to the cell media of cultured human neuroblastoma cells (SH-SY5Y) [25]. Cell viability was evaluated after 72 hours using the MTT or resazurin assays, measuring cellular metabolic activity. The cells were also inspected microscopically before addition of MTT. These inspections were in all cases consistent with the result of the MTT assay.

**Figure 1:**
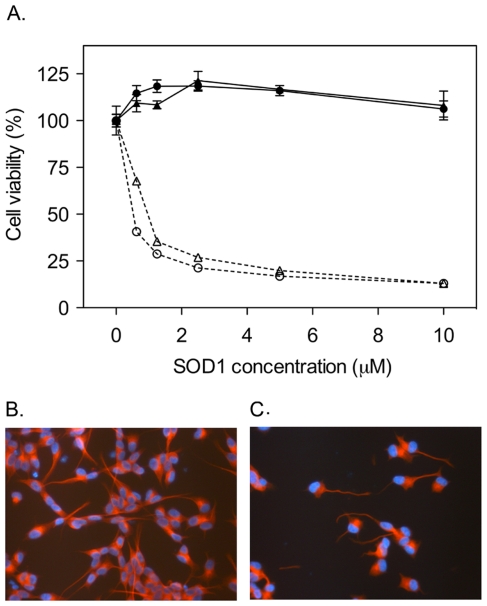
ApoSOD1 reduces viability of cultured cells. Apo and holoSOD1 in dimeric and monomeric form were added to the cell media of cultured SH-SY5Y cells and incubated for 72 h. Proteins were added in duplicate wells and cell viability was measured with the MTT assay. Cell viability is presented as the mean percentage viability of the buffer control ± range. Cell morphology was visualized with immunocytochemistry, using DAPI for DNA staining (blue) and an anti-tubulin antibody for staining of the cytoskeleton (red). (**A**) Both monomeric (○) and dimeric (▵) apoSOD1 induced a cytotoxic response when added in µM concentrations (calculated as monomeric SOD1 concentration for both dimer and monomer). Neither monomeric (•) nor dimeric (▴) holoSOD1 induce toxicity in the same concentration interval. (**B**) Monomeric holoSOD1 (5 µM) did not induce any morphological cell alterations after 72 h of incubation (400× magnification). (**C**) Remaining cells after exposure to 5 µM monomeric apoSOD1 for 72 h displayed an altered morphology characterized by twisted and distorted neurites (400× magnification).

The results show that both monomeric and dimeric apoSOD1 inhibited MTT reduction in the low µM range, demonstrating reduced cell viability, whereas the viability of cells exposed to holoSOD1 remained high (Figure 1A). The same result was observed when using the resazurin assay and in one additional human neuroblastoma cell line (IMR-32) [26], as well as in PC-12 cells from rat pheochromocytoma (Figure S1). To examine more closely the morphology of cells exposed to apoSOD1, SH-SY5Y cells were stained with an anti-tubulin antibody to visualize cytoskeletal alterations. After 72 h of incubation with monomeric apoSOD1, remaining cells displayed distorted and twisted neurites (Figure 1C), whereas cells incubated with holoSOD1 displayed normal morphology (Figure 1B). Images at lower magnification clearly show a reduced number of cells left on the surface after exposure to apoSOD1 (Figure S2).

### The reduced cell viability caused by apoSOD1 does not depend on protein aggregation

**Figure 2:**
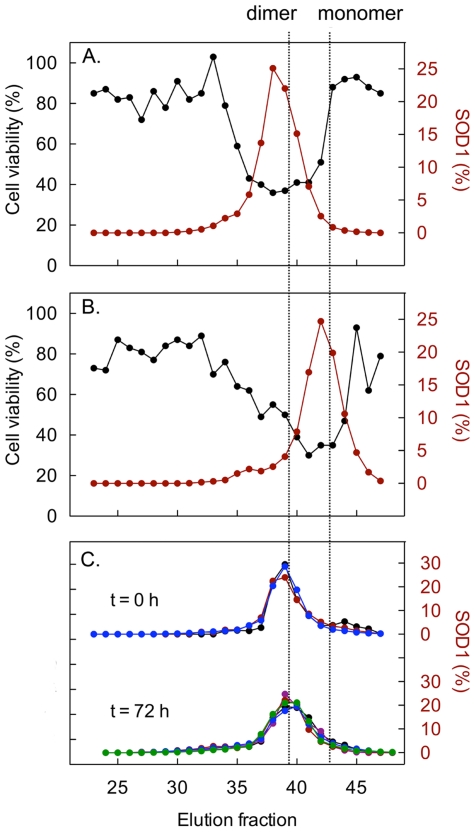
The apoSOD1 molecules do not self-assemble in the cell culture media. Dimeric and monomeric apoSOD (25 µM) were applied to a Superdex 75 column and the eluted fractions were tested for cytotoxicity in SH-SY5Y cells (black) and analysed for SOD1 by western immunoblotting (red). Cell viability was measured with the resazurin assay and presented as percentage viability of buffer control. The peak areas from the western blot analysis have been normalised to total amount of SOD1 in the chromatography. Cytotoxicity coincides with the dimeric (**A**) and monomeric (**B**) peaks of the chromatograms. (**C**) Analysis of the cell-culture medium directly after protein addition of dimeric apoSOD1 and after 72 h incubation confirms that the apoSOD1 molecules remain dimeric throughout the toxicity assay. Large molecules and aggregates would elute in the void volume of the Superdex-75 column around fraction 26. The concentration of SOD1 in the culture medium was varied between 0.16 µM (black), 0.625 µM (purple), 1.25 µM (red), 2.5 µM (blue) and 12.5 µM (green).

Even if apoSOD1 is relatively resistant to spontaneous aggregation *in vitro*, it can readily be provoked to form fibrillar structures upon destabilization or agitation by stirring [23], [27], [28]. As protein aggregation has been associated with reduced viability in cell cultures, both for disease associated [29] and non-disease associated proteins [30], the significance of aggregation for cytotoxicity induced by apoSOD1 was evaluated. Monomeric and dimeric apoSOD1 were analyzed by size-exclusion chromatography, and fractions were collected and subsequently incubated with cells. The fractions exerting high toxicity had an elution volume corresponding to monomeric (Figure 2B) and dimeric (Figure 2A) protein, suggesting that these are the main cytotoxic species. A small shoulder of larger species was observed for the monomeric protein, but these fractions were essentially inert.

To determine the aggregation state of dimeric apoSOD1 in the cell media at the end of the experiments, the collected conditioned cell media were separated by size-exclusion chromatography and the collected fractions were analyzed with western blot. After 72 h of incubation with cells, dimeric apoSOD1 still remained dimeric in a concentration range from 0.16 to 12.5 µM (Figure 2C), demonstrating a toxic response independent of protein aggregation.

### ApoSOD1 toxicity is modulated by mutational changes of protein stability

**Figure 3:**
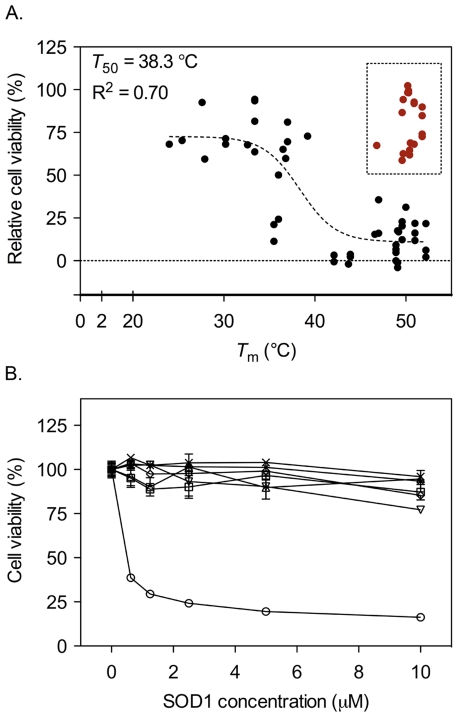
ApoSOD1 with perturbed Zn^2+^ site is non-toxic. A set of apoSOD1 mutants with altered protein stability, charge and Zn^2+^ binding capacity was added to SH-SY5Y cells in duplicate or triplicate in a concentration range of 0.6 to 10 µM and incubated for 72 h. Cell viability was measured with the MTT assay. (**A**) Relative cell viability was calculated as described in methods, and plotted as a function of melting temperature, *T*_m_ (Eq.5, Table 1). A correlation between cell viability and protein stability is observed; overall, the viability is higher for SOD1 mutants with low *T*_m_. SOD1 variants with mutated Zn^2+^ ligands as well as [F64A] and [D124G], all with a diminished ability to bind Zn^2+^, fall outside of this pattern (red symbols) (for detailed information, c.f. Table 1). The line represents a sigmoidal fit (Eq. 5) of all data points, except those from mutants with diminished ability to bind Zn^2+^. (**B**) Monomeric apoSOD1 (○) induces a toxic response, whereas [H63/71/80S;D83S] (□), [H71/80S;D83S] (▵), [H71/80S] (▿) and [H71S] (⋄) are all non-toxic. As all of these mutants lack H71, this amino acid was engineered back into [H63/71/80S;D83S] and [H71/80S;D83S], creating the mutants [H63/80S;D83S] (×) and [H80S;D83S] (✶). However, these mutants show no effect on cell viability, indicating that it is the concerted action of the Zn^2+^ ligands that underlies the toxicity, not H71 alone. Proteins were added in duplicate and data are presented as mean percentage of buffer control ± range.

Earlier studies have shown that apoSOD1 folds by a cooperative two-state process [3], [4],
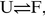
(Scheme 1.)where U and F are the unfolded and folded species, respectively. Accordingly, the relative occupancy of folded material at any given condition is given by the equilibrium constant for folding
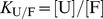
(1)or, commonly, by the protein stability
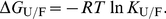
(2)Thus, if the protein stability decreases the occupancy of unfolded protein increases. From analysis of ALS associated SOD1 mutations it is apparent that decreased protein stability is one of the key disease determinants, implicating unfolded, or partly unfolded, protein as the starting material for toxic gain of function [3], [11]. In this study, however, we observe that the toxic responses induced by apo monomers and dimers are of similar magnitudes, despite the higher stability and lower occupancy of denatured material of the apoSOD1 dimer (Figure 1A, Table 1) [4]. Somewhat unexpectedly, this implicates the fully folded apo protein as the principal toxic species. To examine more closely the effect of protein stability on cell viability in this model, we examined the toxic effect of a set of mutants carrying mutations altering protein stability (Table 1). Each mutation was introduced into the monomeric SOD1 protein, and the stability of each mutant was estimated by the thermal melting point using CD spectroscopy (Eq. 3). Cell viability was measured after 72 h of incubation with apo protein in a concentration range of 0 to 10 µM. Viability was expressed as the area under the curve where buffer was set to 100% viability and monomeric apoSOD1 set to 0% (see Methods). When plotting viability as a function of protein stability, a negative correlation was revealed, i.e. unstable mutants were less toxic than mutants with wild-type like stability (Figure 3A). Fit of a sigmoidal transition to the data yields a midpoint of *T*_50_ = 38.3 °C (Eq. 5), close to the temperature under which the experiments were conducted. In other words, mutants with *K*_U/F_ = [U]/[F] = 1 at 37°C, i.e. constructs that are half unfolded in the cell medium, exert half of the cytotoxic effect. Although the uncertainty of this sigmoidal fit is high – the more reductionist fit of a linear correlation is statistically as good - it complies nicely with the observation that folded apoSOD1 is responsible for the cytotoxic action in these experiments. Generally, the mutations with melting temperature below the experimental temperature, i.e. those that are predominantly unfolded in the cell medium, are non-toxic whereas the more stable, folded proteins are toxic. Notably, a toxicity mechanism based on protein aggregation is expected to show the opposite trend. Moreover, the correlation in Figure 3A is opposite to the trend indicated in ALS patients, where decreased protein stability is correlated with reduced survival time [3], [11], [13]; the toxic precursor in ALS patients seems to be unfolded SOD1 material whereas the toxic species in cultured cell seems to be the fully folded apo protein. This questions the disease relevance of the employed cell model. To further elucidate the molecular basis of the toxic effect in cultured cells, another potential disease factor, protein net charge [14], was investigated.

**Table 1:** Melting temperature (*T*_m_) and cell viability of apoSOD1 protein variants.

SOD1 variant	*T*_m_ a. (°C)	Viabilityb. (%)	Min/Maxc.	nd.
C6/111A;F50E;G51E	48.9	0	-	-
C6/111A	54.2	16	-	1
I18V	43.7	(−1.9)	-	1
V29A	30.2	70	68/71	2
V31A	35.5	16	11/21	2
I35V	43.9	3.1	2.3/3.8	2
L38A	22.4	70	-	1
V47A	36.5	65	-	1
F64A	46.8	67	-	1
V81A	47.0	26	16/36	2
L84A	32.6	68	-	1
V87A	39.2	72.8	-	1
V97A	27.6	93	-	1
I104A	37.0	75	70/81	2
L106A	24.0	68	-	1
I112A	36.8	60	-	1
L117A	36.0	37	24/50	2
V119A	27.9	60	-	1
D124G	50.5	69	-	1
L144A	46.6	16	-	1
I149V	42.1	1.3	(−0.6)/3.2	3
C6/57/1111/146A	33.4	83	64/94	3
D11K	49.1	4.1	(−4.0)/18	3
D96K	49.6	19	12/23	3
D109K	52.2	10	2.2/22	3
D11/96K	48.9	6.9	4.8/9.2	3
D11/96/109K	51.0	17	12/22	3
D11/92/96/109S;E24/121S	50.0	31	-	1
H46/48/120S	49.2	17	-	1
H63/71/80S;D83S	51.8	80	73/90	4
H71/80S;D83S	50.9	84	68/93	3
H71/80S	49.6	73	59/87	2
H71S	49.7	78	63/94	2
H63/80S;D83S	50.2	100	98/102	2
H80S;D83S	50.3	99	98/99	2
H46/48/63/71/80/120S;D83S	50.4	63	62/65	2

a. The midpoint of the thermal unfolding transition monitored by CD, as derived by fitting of a sigmoidal function (Eq. 3), using a Δ*C*_p_ value of 1.4 kcal mol^−1^ K^−1^. This value was derived from the slope of Δ*H*_U/F_(T_m_) vs. T_m_ for the first 20 mutants in Table 1 (data not shown).

b. Mean cell viability expressed as percentage of buffer control with monomeric apoSOD1 [C6/111A;F50E;G51E] set to 0%. Replicate values are displayed in Figure 3A.

c. Min/Max represents the minimum and maximum viability response observed for the individual mutations.

d. Number of cell viability experiments for the individual SOD1 mutations.

### Alteration of protein net charge has no effect on cell viability

Reduced net negative charge has previously been identified as a disease-provoking property among ALS-associated SOD1 mutations [14]. To study the effect of protein net charge on cell viability in the model presented herein, the net charge of monomeric SOD1 was increased by the mutations D11K, D96K and D109K, individually or as double or triple mutants (c.f. Table 1). These mutations alter the net charge of the protein from -8 to -6, -4 and -2 at neutral pH (as calculated directly from the amino-acid sequence at pH 7, assuming that the histidines are uncharged). In addition, we produced a control mutant with six negatively charged amino acids substituted for serines, [D11/92/96/109S; E24/121S]. This control carries the same net charge as the [D11/96/109K] triple mutant but with no increase in the number of positive amino acids. The thermodynamic stability of these charge mutants was virtually identical to that of the monomeric wild-type protein (Table 1). The results show that alteration of the net negative charge by substituting D for K did not significantly alter the toxic response. Also, the mutant [D11/92/96/109S; E24/121S] retained a toxicity close to that of the wild-type protein (Table 1). Again, the toxic response observed with cultured cells does not follow the pattern of ALS-associated SOD1 mutations.

### Demetallation of monomeric apoSOD1 increases flexibility of loops IV and VII

**Figure 4:**
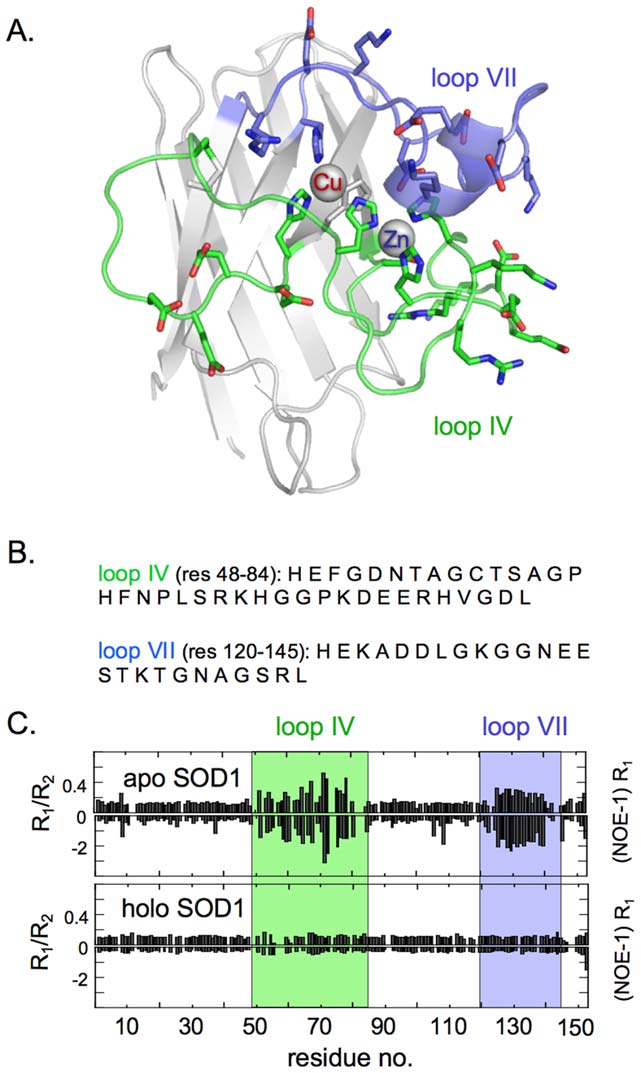
Loss of Cu^1+/2+^ and Zn^2+^ ions leads to increased backbone dynamics of the active-site loops IV and VII. The backbone dynamics of monomeric apo- and holoSOD1 evaluated by NMR relaxation experiments. (**A**) Structural representation of the SOD1 monomer showing the regions of increased dynamic motions in the apo state. (**B**) Amino-acid sequence of loops IV and VII. (**C**) Increased dynamics in the intermediate time scale is indicated by increased values of R_1_/R_2_ and increased fast dynamics as increased negative values of (NOE-1)R_1_. In the apoSOD1 monomer, the regions of loops IV and VII show increased dynamics [31], whereas in the metallated monomer [71] the loops are as fixed as the rest of the protein. Figure outline adapted from Nordlund *et al.* [32].

Demetallation of SOD1 have previously been observed to increase the dynamic motions of loops IV and VII [31], [32], pointing at the possibility that these parts of the structure are involved in the cytotoxic action of the apo protein. The augmented dynamics of loops IV and VII were confirmed for the monomeric apoSOD1 species in this study, using R_1_, R_2_ and heteronuclear NOE NMR relaxation experiments [33], [34]. The results show that monomeric holoSOD1 undergoes overall small and uniform dynamic motions in loops IV and VII (Figure 4). This restricted flexibility is revealed by the uniformly low and even values of R_1_/R_2_ and (NOE-1)R_1_, which are typical for well ordered, globular proteins [35]. Upon removal of the metal ions, however, loops IV and VII undergo marked, local changes seen as a simultaneous increase in the R_1_/R_2_ and (NOE-1)R_1_ values, i.e. the regions of loops IV and VII gain dynamic mobility and become more flexible (Figure 4). Accordingly, the gain of cytotoxic function upon demetallation seems to coincide with increased loop dynamics. Since loops IV and VII are the only regions to show structural differences upon demetallation, it is further implicated that this part of the SOD1 structure is directly coupled to the gain of cytotoxic function in the cultured-cell system.

### Mutation of the Zn^2+^-binding ligands abolishes the cytotoxic effect of apoSOD1

From a functional perspective, the active-site loops structures in SOD1 play a key role by binding the Cu^1+/2+^ and Zn^2+^ ions. The Zn^2+^ ion is coordinated by three histidines, H63, H71 and H80, and one aspartic acid, D83, all located in loop IV. H63 also coordinates Cu^1+/2+^ together with H46, H48 and H120. To investigate the role of Zn^2+^ binding and the Zn^2+^-binding side chains in the cytotoxic action of SOD1, we produced the mutations [H63/71/80S;D83S], [H71/80S;D83S], [H71/80S] and [H71S]. Notably, all of these variants with mutated Zn^2+^ ligands show up as clear outliers in the stability vs. cell viability plot (Figure 3A, within square): they are virtually non-toxic even though they show wild-type like stability and are well folded under the experimental conditions. In contrast, the apo state of the Cu^1+/2+^-ligand mutant [H46/48/120S], which has retained affinity for Zn^2+^ [36], induces a toxic response of a magnitude that is expected from its protein stability (Table 1). Conversely, the SOD1 variants [F64A] and [D124G], both with a reduced affinity for Zn^2+^ (Figure S3, [37]), fall outside of what can be expected from protein stability alone (Table 1). As the Zn^2+^-ligand mutants all lack the residue H71, this amino acid was engineered back creating the new variants [H63/80S;D83S] and [H80S;D83S]. However, this modification had no discernable effect on the cell viability (Figure 3B), demonstrating that the toxic effect is not mediated by this single amino acid alone. Rather, the cytotoxicity seems to require an intact high-affinity Zn^2+^ site. As a further test of the toxicity mechanism, we demonstrated that the apoSOD1 toxicity could not be inhibited by heparin, generally expected to compete with heparan sulphate binding sites (Data not shown). Thus, the cytotoxicity observed in this study is unlikely related to interaction of Zn^2+^-binding residues with heparan sulphate as observed for the Aβ peptide implicated in Alzheimer's disease [38], [39].

### The apoSOD1 toxicity builds up over time and can be halted by Zn^2+^ addition

**Figure 5:**
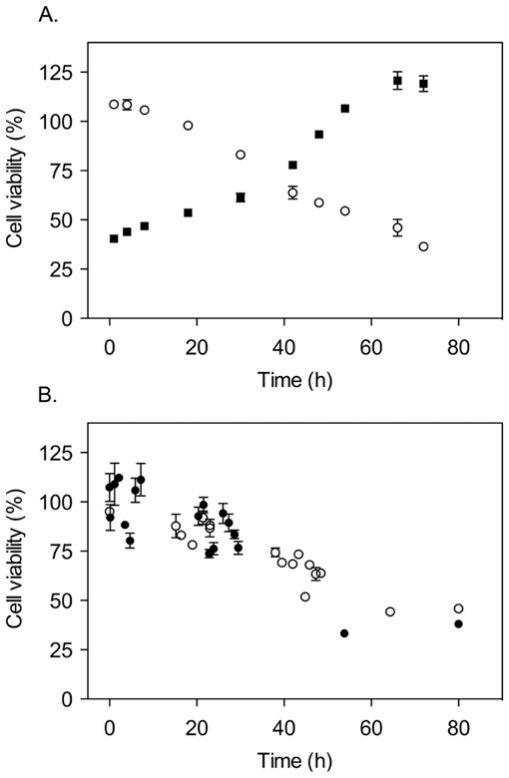
ApoSOD1 toxicity builds up over time and can be halted by Zn^2+^ addition. 10 µM monomeric apoSOD1 was first added to multiple wells of SH-SY5Y cells and then washed away after different times of incubation. In parallel to washing, 10 µM monomeric apoSOD1 was also added to untreated cells. In a second line of experiments, 10 µM monomeric apoSOD1 [H46/48/120S] was added to the cells and, after different times of incubation, 20 µM of ZnCl_2_ was added. MTT was added at the endpoint (72 h) of both experiments. Proteins were added in duplicate and data is presented as mean percentage of the buffer control ± range. (**A**) Washing away the apoSOD1 protein after different length of incubation (○) induces the same toxicity as when adding the protein the same number of hours before the endpoint of the experiment (▪). (**B**) Adding Zn^2+^ to the cell media after different delay times has the same effect on cell viability as washing the protein away. Filled and empty symbols represent separate experiments.

To examine the time dependence of the cytotoxic response, two approaches were attempted to neutralize the cytotoxic apoSOD1 protein; (i) washing off the protein from the cells by exchanges of the culture media and (ii) addition of Zn^2+^ ions to the culture media. The results showed that washing off the monomeric apoSOD1 protein after less than 30 h of incubation completely saved the cells. In a parallel control experiment, where the addition of apoSOD1 was delayed after the start of the incubation, it was further demonstrated that 30 h incubation with the protein is the time needed for cell death to commence (Figure 5A). These observations indicate that the reduced viability caused by apoSOD1 is integrated - builds up - over time, as opposed to an early decisive event that predisposes the cells for death. Consistently, when Zn^2+^ was supplied to the culture media within 30 h after the addition of apoSOD1 [H46/48/120S] the cells were also saved, following the same time dependence as observed when apoSOD1 was washed off from the cells (Figure 5B). No other metals tested, including Cu^2+^ and Fe^2+/3+^, saved the cells from cytotoxicity (Figure S4). Zn^2+^ reduced the toxicity almost completely (80% viability) at equimolar concentration, indicating that Zn^2+^ binding to free apoSOD1 in the culture medium stops Zn^2+^ depletion of the cells (Figure S4A). In a second experiment, apoSOD1 was added at a concentration where viable cells are still remaining at the endpoint of the experiment (32% viability). None of the metals added in excess concentration increased toxicity (Figure S4B), suggesting that these metals do not interact with apoSOD1 in such a way that free radicals or other noxious processes emerge. These data, again, point at the ability of apoSOD1 to coordinate Zn^2+^ as the key determinant for the toxic effect.

### Reduced cell viability exerted by apoSOD1 is not dependent on serum

To examine if apoSOD1 induces toxicity through alteration of serum components, the effect of monomeric apoSOD1 on cell viability was evaluated also under totally serum-free conditions. The results show that the toxicity is not dependent on serum: the toxic effect of apo SOD1 remains under serum-free conditions (Figure S5).

### Addition of Zn^2+^ chelators induces a toxic response similar to that of apoSOD1

**Figure 6:**
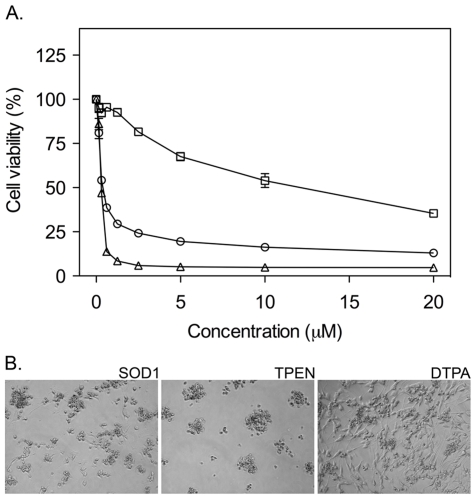
Zn^2+^ chelators induce a toxic response similar to that of apoSOD1. The high-affinity Zn^2+^ chelators TPEN and DTPA were dissolved in DMSO and added to SH-SY5Y cells in parallel with monomeric apoSOD1 in a concentration range of 0.16 to 20 µM, and incubated for 72 h before addition of MTT. The final concentration of DMSO in the cell culture was 0.1%. (**A**) TPEN (▵) induces a toxic response similar to that of monomeric apoSOD1 (○), whereas DTPA (□) shows an overall weaker effect. Chelators and apoSOD1 protein were added in duplicate and data are presented as mean percentage of vehicle control ± range. (**B**) Phase contrast images of cells exposed to 10 µM monomeric apoSOD1, 10 µM TPEN and 10 µM DTPA. Cells incubated with apoSOD1 closely resemble cells incubated with TPEN.

One explanation for the requirement of un-metallated, high-affinity Zn^2+^ sites for the apoSOD1 cytotoxicity is Zn^2+^ chelation. The level of free Zn^2+^ ions in the cytoplasm is regulated through a system of proteins buffering Zn^2+^ and transporting Zn^2+^ in and out of the cell [40]. Disturbances in this balance can be deleterious [41]. Of particular interest for this study is that the addition of Zn^2+^ chelators is previously reported to have pronounced effects on the viability of cultured cells [42], [43], [44]. Even if TPEN and DTPA show high affinity for other divalent cations, their cell toxicity have in several studies been attributed to the specific chelation of Zn^2+^; in essence, their cytotoxic effect is preferentially inhibited by addition of Zn^2+^ ions [43], [45], [46], [47]. To compare the toxic response of apoSOD1 with that of high-affinity Zn^2+^ chelators, we incubated monomeric apoSOD1 in parallel with equimolar amounts of N,N,N′,N′-Tetrakis(2-pyridylmethyl)ethylenediamine (TPEN, membrane permeable) and Diethylene triamine pentaacetic acid (DTPA, membrane impermeable). The results show that apoSOD1 was slightly less toxic than TPEN, but considerably more toxic than DTPA (Figure 6A). However, the reason for this difference is not necessarily related to the limited membrane permeability of DTPA, but can also arise from competing uptake of other divalent ions, e.g. Ca^2+^, present at high concentrations (mM) in the culture media. Thus, the relatively low toxicity of DTPA could simply be due to partial saturation by Ca^2+^ [44], [48], [49], which reduces the chelator's capacity to scavenge free Zn^2+^. Moreover, we observe that phase-contrast images of cells exposed to TPEN agree with the cell viability data by displaying cells with a rounded-up appearance, closely resembling cells incubated with apoSOD1 (Figure 6B). Together with the similar effect of apoSOD1 and TPEN on the cell viability as measured by the MTT assay, this observation supports the idea that the toxicity mechanism of apoSOD1 in this model involves Zn^2+^ chelation.

### Zn^2+^ is transferred from the cells to the apoSOD1 protein

To examine if intracellular Zn^2+^ is actually transferred from the cells to the SOD1 protein during the cytotoxic action, cells were loaded with the radioactive isotope ^65^Zn by incubating the cells in cell media containing ^65^Zn. After washing and replating the cells under serum-free conditions, 10 µM of monomeric apoSOD1 [C6/111A;F50E;G51E] and the mutants [H46/48/120S], [H71/80S;D83S] and [H46/48/63/71/80/120S;D83S] were added to the culture media. The monomeric apoSOD1 [C6/111A;F50E;G51E] protein was determined by ICP-MS to initially contain 0.03 mol Zn^2+^/mol SOD1 (analyzed by ALS Scandinavia, Sweden).

**Figure 7:**
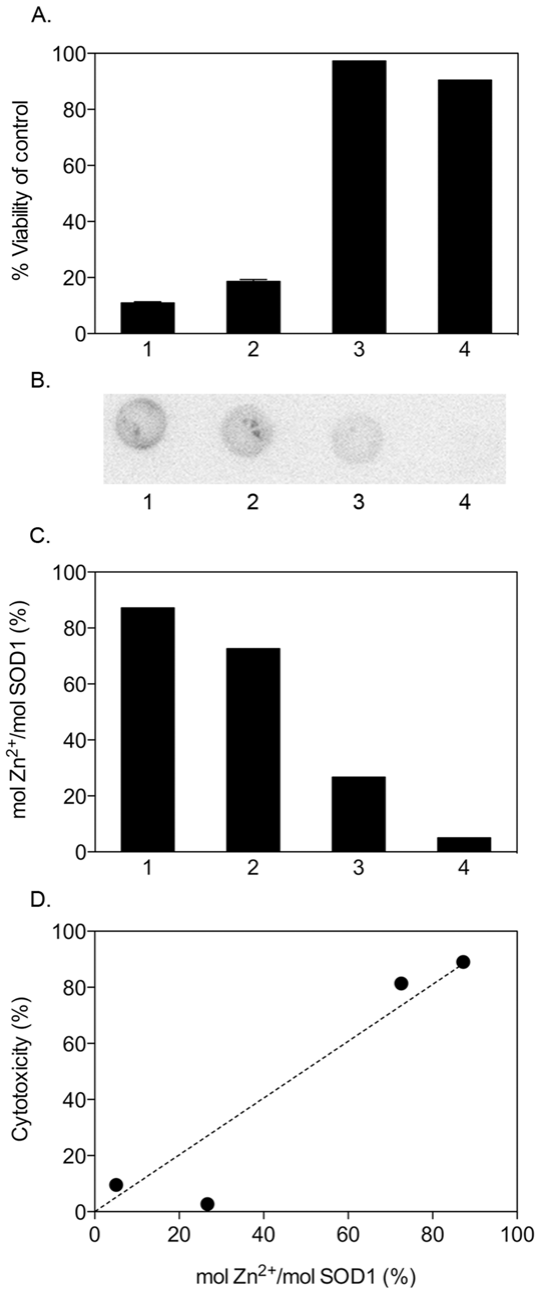
Intracellular Zn^2+^ is transferred to cytotoxic apoSOD1 proteins. Monomeric apoSOD1 [C6/111A;F50E;G51E] (**1**) and the mutants [H46/48/120S] (**2**), [H71/80S;D83S] (**3**) and [H46/48/63/71/80/120S;D83S] (**4**) were added to a final concentration of 10 µM to SH-SY5Y cells loaded with radioactive ^65^Zn. Samples of the culture media were collected, concentrated dry on a 10 K filter and exposed to a phospho-imaging screen. As reference for signal quantification, we used lysate of ^65^Zn-loaded cells and a standard series of known ^65^Zn concentrations. Cell viability was measured in a parallel experiment. (**A**) Monomeric apoSOD1 [C6/111A;F50E;G51E] and [H46/48/120S], which both show high Zn^2+^ affinity, reduce cell viability to below 20%, whereas the low Zn^2+^ affinity mutants [H71/80S;D83S] and [H46/48/63/71/80/120S;D83S] have no significant effect on cell viability. (**B**) Filter radioactivity showing transfer of ^65^Zn to the different SOD1 variants in panel A. The signal intensity declines with decreasing Zn^2+^ affinity of the apoSOD1 proteins concentrated on the filter. The high-density patterns in 1 and 2 result from manufactured depressions in the filter surface. (**C**) The molar ratio of Zn^2+^ to SOD1 decreases with the Zn^2+^ affinity of the protein. 100% is the ratio expected if all ^65^Zn present in the cells (quantified to 1.9 fmol/100 000 cells) would transfer to the protein. (**D**) Plot of cytotoxic response (1-viability %) vs. molar ratio of Zn^2+^ to SOD1.

After 70 h of incubation, culture media samples were collected, concentrated dry on cellulose filters and analyzed for ^65^Zn content by exposing the filters to a phosphoimaging screen together with a standard series of known ^65^Zn concentrations applied to nitrocellulose. Sample handling times were kept as short as possible, i.e. less than 1.5 h, to minimise ^65^Zn loss from the protein by dynamic breathing [36]. After 70 h of incubation, cell viability was significantly reduced for cells incubated with the toxic apoSOD1 [C6/111A;F50E;G51E] and [H46/48/120S], whereas cells incubated with the non-toxic [H71/80S;D83S] and [H46/48/63/71/80/120S;D83S] were fully viable (Figure 7A). The toxic apoSOD1 [C6/111A;F50E;G51E] and [H46/48/120S] contained 87% and 73% ^65^Zn, respectively (Figure 7C), where 100% is the level if all ^65^Zn available in the cells (quantified to 1.9 fmol/100 000 cells) would transfer to the protein. As expected, the ratio of ^65^Zn bound to the non-toxic proteins [H71/80S;D83S] and [H46/48/63/71/80/120S;D83S] were significantly lower at 27% and 5%, respectively. The results show that the cytotoxicity in this study is accompanied by transfer of intracellular Zn^2+^ ions to extracellular apoSOD1, consistent with our interpretation above. Finally, and independent of the absolute quantification of ^65^Zn, the relatively lower ^65^Zn contents of the non-toxic mutants with decreased Zn^2+^ affinity support the conclusion that the cytotoxic action of apoSOD1 is linked to the uptake of Zn^2+^ ions.

## Discussion

### Cytotoxicity induced by extracellularly added SOD1 is independent of aggregation

Even though SOD1-positive inclusions are observed both in familial [50] and sporadic ALS [51], [52], as well as in transgenic mice [53], SOD1 lacking C6 and C111 stands out as a relatively soluble protein *in vitro*. Aggregation needs to be triggered by agitation under reducing or otherwise destabilizing conditions [5], [28]. Consistently, no SOD1 aggregates could be detected at the endpoint of our cell experiments (Figure 2C). Instead, the reduced cell viability arose from the chromatographic fractions containing soluble apoSOD1 monomers (Figure 2B) and dimers (Figure 2A). It is thus apparent that the cytotoxicity of apoSOD1 in this study does not rely on protein aggregation. This observation contrasts studies of other precursors of neurodegenerative diseases, e.g. Aβ [29], α-synuclein [54] and transthyretin [55], where aggregation is implicated as the prime cause of toxicity in cell cultures. Moreover, the coupling between decreased cell viability and high apoSOD1 stability (Figure 3A) indicates the folded protein as the cytotoxic species. A similar correlation has been reported for the protein Onconase A [56], which is an RNaseA homolog with specific cytotoxic activity against cancer cells. In the case of Onconase A, however, the cytotoxicity depends on the protein's ability to evade proteolysis [56]. A yet different toxicity mechanism has been suggested for a microbial RNaseA, which stems from interaction with negative glycolipids in the plasma membrane. Characteristic to this type of mechanism is that it depends critically on the proteins net charge: an increase of the global charge of RNaseA from −7 to +3 was found to decrease the toxicity drastically [57]. In the case of SOD1, however, we observe no corresponding effect of changing the global charge from −8 to −2 (Table 1), suggesting that the cytotoxic mechanism is of different origin. Judging by the NMR analysis (Figure 4) and the ability of Zn^2+^ to inhibit cytotoxicity (Figure 5B), the action of apoSOD1 seems rather linked to the structural properties of the active-site loops IV and VII.

### ApoSOD1 toxicity requires empty, high-affinity Zn^2+^ sites

From the data in Figure 3 it is clear that several apoSOD1 mutants deviate from the viability vs. stability plot. Common to these outliers is that they lack toxicity even though they remain folded in the culture media. Common to the outliers is also that they affect the Zn^2+^-binding capacity of the apoSOD1 molecule. Substitution of only one Zn^2+^-binding ligand (i.e. H71) is enough to render the protein non-toxic (Figure 3B). This, in combination with the lack of toxicity of holoSOD1 (Figure 1A, 1B) and the inhibitory effect of Zn^2+^ supplementation (Figure 5B), but not other metals (Figure S4), on the apoSOD1 toxicity, points at the actual uptake of Zn^2+^ as the prime cause of cytotoxic function. Without exception, the mutants that cannot coordinate Zn^2+^ with sub µM affinity have no effect on cell viability and cluster in the top-right corner of the viability vs. protein stability plot (Figure 3A). Consistently, alterations of the neighbouring Cu^1+/2+^ ligands, which have only small influence on the Zn^2+^ affinity, have no corresponding effect on cell viability: the mutant [H46/48/120S], which completely truncate the native Cu^1+/2+^ site, exerts perfectly wild-type like toxicity (Table 1). Taken together, this toxicity pattern does not comply with the earlier observations by Estevez *et al.* where the toxicity of Zn^2+^-deficient SOD1 is dependent on the redox-active Cu^1+/2+^ ion, allowing the production of noxious peroxynitrite radicals [20]. Also, the inability of Cu^1+/2+^ and Fe^2+/3+^ to increase cytotoxicity (Figure S4B) argues against the involvement of a redox-active metal interacting with the Zn^2+^ site. The toxicity observed in this study seems instead to rely on the apoSOD1 molecule's ability to bind Zn^2+^.

### Toxicity by Zn^2+^ chelation

As the apoSOD1 cytotoxicity seems to rely on Zn^2+^ affinity, Zn^2+^ chelation stands out as the most reductionist and plausible underlying mechanism. Given a Zn^2+^ level of 0.4 fmol/cell [58], the Zn^2+^ content in the culture wells provided by the cells (30000 cells in a culture volume of 100 µl) corresponds to 120 nM. The background level of Zn^2+^ in the culture media with 0.5% serum, as used in these experiments, is estimated to 200 nM based on known Zn^2+^ levels in supplemented media with 10% serum [59]. This Zn^2+^ content is well within the binding range of the µM apoSOD1 levels observed to induce toxicity in this study; the Zn^2+^ affinity for SOD1 is <nM [60], [61]. In direct support of this idea, low molecular weight Zn^2+^ chelators have in several studies been shown to be toxic to cultured cells [42], [43], [62]. In the simplest case, chelation of Zn^2+^ is deleterious because several essential proteins depend critically on Zn^2+^ for their function. One example is coordination of Zn^2+^ to the active site of histone deacetylases, a class of enzymes that control DNA integrity and gene expression [63]. Inhibitory binding of small organic molecules to the Zn^2+^ site of this protein has even emerged as a promising strategy in cancer therapy [64], [65].

**Figure 8:**
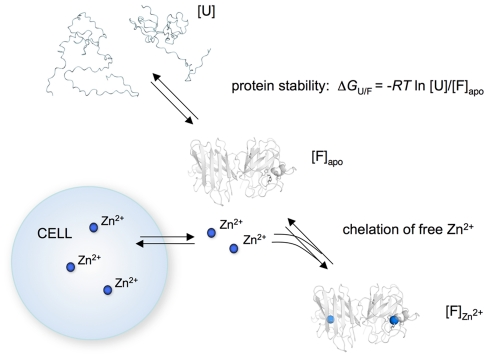
Schematic outline of how apoSOD1 exerts toxicity to cultured cells. Protein stability (Δ*G*_U/F_) determines the relative concentrations of unfolded ([U]) and folded ([F]) apo protein and, thus, the effective concentration of species able to chelate Zn^2+^ from the growth medium. For stable mutations where [F]_apo_≫[U], the protein acts as an efficient Zn^2+^ chelator, disturbing the cellular Zn^2+^ homeostasis. In the most simplistic case, the apoSOD1 molecules do not interact with the cells but lower the intracellular Zn^2+^ levels by preventing reflux from the growth medium.

In striking resemblance with the observations in the present study, extracellular Zn^2+^ depletion is implicated in the toxic action of calprotectin, a Ca^2+^- and Zn^2+^-binding protein that reduces cell viability in a Zn^2+^-reversible manner [66]. A distinct feature of the calprotectin toxicity is that it does not require direct cell contact: depleting Zn^2+^ from the surrounding growth medium appeared to be enough. Thus, it is reasonable to assume that the apoSOD1 toxicity can also be exerted without direct cell contact (Figure 8).

### Concluding remarks

The reductionist conclusion from this study would be that the observed cytotoxicity based on Zn^2+^ chelation constitutes an artefact of an overly simplistic cell model, rather than a true component of the ALS mechanism. Nevertheless, since cultured cells represent one of the most commonly employed toxicity models in neurodegenerative disease, such an inherent sensitivity to the Zn^2+^ levels could have critical implications for the interpretation of data. Not only in studies of metalloproteins, but also in studies of protein aggregates in general, which are known to coordinate ions in a more unspecific manner [67]. The corresponding effects of chelating Zn^2+^ in intact neural tissue, and whether such perturbations play any role in protein-aggregation disease, are yet to be found out. There is, however, increasing evidence for the importance of Zn^2+^ in synaptic transmission and plasticity, as well as the importance of maintained Zn^2+^ homeostasis in cell survival [58], [68]. It is therefore conceivable that apoSOD1, if secreted or transferred to the extracellular space by cell lysis, could modulate the propagation of neuronal damage in ALS [6]. Dietary Zn^2+^ supplementation has also been shown to be protective in G93A mice [69]. Still, based on the data at hand, the apoSOD1 toxicity mediated by Zn^2+^ chelation is most likely limited to the cell model of this study and not the trigger of human ALS. From a methodological standpoint, our results show also that rational protein engineering in combination with biophysical analysis can be used to produce protein specific data that correlate quantitatively with the cellular response: free-energy perturbation is a powerful tool for mapping out molecular mechanism also in cellular systems.

## Methods

### Protein expression and purification

To ensure good metal loading, SOD1 was co-expressed with the yeast Cu-chaperone CCS in E.Coli. The bacterial cultures were grown at 23°C, and CuSO_4_ (3 mM) and ZnSO_4_ (30 µM) were added upon induction. Purification was done by heat denaturation (55°C for 30 min) followed by ammonium sulfate precipitation, gel filtration (S100 Sephacryl 100, Amersham Pharmacia), and ion-exchange chromatography (Q-Sepharose) [11]. The metal content for the monomeric holo protein was determined to 80% zinc and 20% copper (*data not shown*), using Total Reflection X-ray Fluorescence (TXRF).

### Apo procedure

Apo protein was prepared by adding the protein solution to a Slide-a-Lyzer mini dialysis tube (Pierce) placed in a solution of 4 M of guanidinium chloride, 10 mM of MES buffer and 250 mM of EDTA for a minimum of 4 h at room temperature. The protein was subsequently dialyzed against 10 mM MES buffer. The apo state of the protein was verified by standard folding assays (chevron plots) [4], [11] and melting curves as described in the Methods section. Any traces of holoSOD1 or misfolded protein would appear as extra phases/transitions, added to those of the apo species. In addition, a sample of monomeric apoSOD1 was analyzed with ICP-MS (ALS Scandinavia, Sweden), and determined to contain a molar fraction of 0.03 Zn^2+^.

### Cell cultures

Human neuroblastoma SH-SY5Y cells were cultured in Dulbecco's Modified Eagle Medium (D-MEM, Gibco) supplemented with 10% FBS, 100 units/ml penicillin, 100 µg/ml streptomycin, 250 µg/ml amphotericin B and 5 µg/ml plasmocin, at 37°C in a humidified atmosphere of 5% (v/v) CO_2_/air.

### 3-(4,5-dimethylthiazol-2-yl)-2,5-diphenyltetrazolium bromide (MTT) assay

SH-SY5Y cells were detached with trypsin and seeded in D-MEM without phenol red supplemented with 1% FBS at a density of 57 000 cells/cm^2^ in 96-well plates and grown over night. Prior to treatment, serumfree D-MEM was added resulting in a final serum concentration of 0.5%. SOD1 protein was incubated with the cells for 72 h, and cell viability was assessed at the end of the experiment by addition of MTT in a final concentration of 5 mg/ml and further incubation for 4 h. The formazan product was dissolved with 20% SDS in 50% dimethyl formamide, and absorbance measured at 570 nm (Spectramax 340 PC). This endpoint measurement determines the total amount of viable cells at the end of the experiment, both adherent cells and possible floaters.

### Resazurin assay

SH-SY5Y cells were detached with trypsin and seeded in supplemented MEM (c.f. Text S1) without phenol red at a density of 40 000 cells/cm^2^ in 96-well plates and incubated for 24 h. Prior to addition of SOD1, the media was exchanged for MEM without serum. SOD1 protein was incubated with the cells for 72 h, and cell viability was assessed by addition of resazurin in a final concentration of 0.01 mg/ml to each well and further incubation for 3 h. Fluorescence was measured using a Tecan Infinity fluorescence microplate reader with excitation at 535 nm and emission at 595 nm.

### Cell imaging

SH-SY5Y cells were plated at a density of 40 000/cm^2^ in lab tek II slides (Nunc) and fixed in 4% (w/v) paraformaldehyde. Unspecific binding sites were blocked in 10% goat serum, and microtubuli were visualized using an anti-β-tubulin antibody (Cell Signalling) and a rhodamine labelled secondary antibody (Rockland). The slide was mounted with Vectashield mounting medium containing 1 µg/ml DAPI (Vector Labs) to visualize nuclei and examined in an inverted Zeiss Axiovert 40 CFL microscope equipped with epifluorescence and a digital Aciocam ICc1 camera.

### Test of SOD1 aggregation by size-exclusion chromatography

Monomeric and dimeric apoSOD1 protein was separated on a Superdex 75 10/300 column (GE Biosciences) equilibrated with MEM without phenol red. The eluted fractions were supplemented with Non-Essential Amino Acids solution (NEAA), 2 mM L-glutamine, 100 units/ml penicillin, 100 µg/ml streptomycin, and 1 mg/ml bovine serum albumin prior to incubation with SH-SY5Y cells. For test of the aggregation state of dimeric apo SOD1 at the endpoint of the viability assay, the samples were centrifuged at 1000 G for 10 min to remove non-adherent cells prior to the chromatography.

### Immunoblotting

For western immunoblots an antibody raised against a peptide corresponding to residues 24–39 in the human SOD1 sequence was used as previously described in Jonsson *et al.* [70]. For quantification, chemiluminescence were recorded with a ChemiDOC XRS (Bio-Rad, Hercules, CA, USA) and analyzed with Quantity One software (Bio-Rad). SOD1 in each fraction is given as percentage of total eluted SOD1.

### CD spectroscopy

The melting temperature (*T*_m_) of each apoSOD1 mutant was determined by CD spectroscopy using a Jasco J-815 equipped with a peltier temperature controller. Proteins were dialyzed against phosphate buffer pH 7.5 (alternatively diluted in the same buffer) before analysis. Thermal denaturation was achieved by increasing the temperature (T) from 5 to 95°C with a rate of about 1°C/min. At each temperature increment, the CD signal (θ) was integrated between 222 and 236 nm. Assuming a first-order unfolding transition between folded (F) and unfolded (U) protein, the resulting plot of θ vs. T was fitted by the sigmoidal function
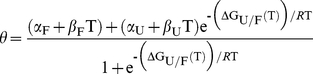
(3)where Δ*G*_U/F_(T) is the protein stability (Eq. 4), and (*α*_F_+*β*_F_T) and (*α*_U_+*β*_U_T) are the base lines for the folded and unfolded states, respectively.
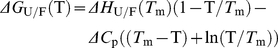
(4)where Δ*H*_U/F_(*T*_m_) is the absorbed heat upon unfolding (i.e. melting enthalpy), *T*_m_ is the melting temperature and Δ*C*_p_ is the heat capacity difference between U and F.

### NMR spectroscopy

Standard T_1_, T_2_ and steady-state heteronuclear NOE experiments were performed on a Bruker 600 MHz spectrometer (Bruker Avance, Karlsruhe, Germany) equipped with a room temperature triple-resonance probe. All experiments were done at 25°C and pH 6.3 with 220–350 µM SOD1. In holo SOD1, Cu^2+^ was substituted with Zn^2+^. Spectra were transformed using nmrPipe and analyzed by Sparky (T. D. Goddard and D. G. Kneller, SPARKY 3, UCSF). In the T_1_ and T_2_ experiments the signal attenuation from 10 different relaxation delays was fitted to a single-exponential decay.

### Normalization and fitting of cell viability data

To have a measure of the total viability response of each mutant in Figure 3A, the area under each viability curve was calculated using Graphpad prism 5.0 and normalized against buffer and monomeric apoSOD1 within each individual experiment using the following expression: (area_mutant_-area_apoSOD1_)/(area_buffer_-area_apoSOD1_). In this way, buffer was set to 100% viability and monomeric apoSOD1 to 0% viability. The dataset was subsequently fitted to a sigmoidal function, describing how the total cell viability response (Y) depends on the melting temperature (*T*_m_) of the individual protein mutants,
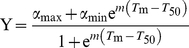
(5)where α_min_ is the bottom base line, α_max_ the upper base line and *T*_50_ is the *T*_m_ value at which the viability has been reduced to 50%, i.e. the transition midpoint. The value *m* defines the width of the sigmoidal transition between α_max_ and α_min_. Data was fitted with graphpad prism v.5.0.

### Loading of cells with ^65^Zn

SH-SY5Y cells were incubated with 200 kBq of ^65^ZnCl_2_ (theoretical specific activity: 305 GBq/mg ^65^Zn) (Perkin-Elmer, 45 MBq/mg total Zn^2+^) in 10 ml of supplemented cell medium for 72 h. Radioactive medium was removed, and the cells were washed 2× with PBS and 1× with 5 mM EDTA in PBS. Cells were detached and plated in serum free medium (100 000/well for 96 well plate, 570 000/well for 24 well plate). 10 µM of apoSOD1 was added and incubated with the cells for 70 h, after which samples of culture media was collected from the 24 well plate, and cell viability measured on the 96 well plate using the MTT assay.

### Cellular uptake of ^65^Zn

1×10^6^ cells loaded with ^65^Zn were pelleted at 1500× g for 5 min and the supernatant removed. The cell pellet was lysed in 100 µl cold cell lysis buffer (50 mM Tris-HCl 150 mM NaCl pH 7.5, 1% NP-40, protease inhibitor cocktail) and incubated for 5 min at 4°C. Cell debris was pelleted at 13 000× g for 10 min at 4°C, and the supernatant was collected and applied to a nitrocellulose membrane.

### Filter assay and quantification of ^65^Zn transfer to apoSOD1

The collected samples of culture media were centrifuged at 1500× g for 5 min, and 500 µl of the supernatant was concentrated dry on a 10 K cellulose filter at 14 000× g for 1 h using mini concentration tubes (Millipore). The amounts of radioactive ^65^Zn in the filters were determined by phospho-imaging. As references for quantification, we used cell lysate and a standard series of known concentrations of ^65^Zn applied to nitrocellulose. The phospho-imaging detection matrix was scanned (Fuji FLA-3000) and pixel intensities were quantified using the MultiGauge software (Fujifilm). The calculations of ^65^Zn/SOD1 stoichiometry were based on 10 µM SOD1 (measured by absorbance at 280 nm), i.e. the concentration of SOD1 in the culture media during the toxicity experiment. According to the chromatographic analysis in Figure 2C the concentration of SOD1 in the culture medium remains approximately constant during the toxicity experiment.

## Supporting Information

Figure S1 **Dimeric apoSOD1 is cytotoxic to three different cell lines.** Dimeric apoSOD1 was added to three different cell lines in a final concentration of 5 µM and incubated for 72 h. Cell viability was measured using the resazurin assay. Proteins were added in triplicate and data are presented as mean ± SD as percentage of the buffer control. ApoSOD1 (white bars) reduced cell viability in all three cell lines, whereas holoSOD1 (grey bars) was non-toxic.
(TIFF)

Figure S2 **Fluorescence microscopy of cells exposed to apo and holoSOD1.** Cells were visualized with immunocytochemistry, using DAPI for DNA staining (blue) and an anti-tubulin antibody for staining of the cytoskeleton (red). (**A**) Monomeric holoSOD1 (5 µM) does not cause any visible cell death after 72 h of incubation (200× magnification). (**C**) Few cells remain after exposure to 5 µM monomeric apoSOD1 for 72 h (200× magnification).
(TIF)

Figure S3 **The thermal transition of apoSOD1 F64A is not affected by Zn^2+^.** Zn^2+^ was added in 5 to 7 molar excess to a protein solution of monomeric apoSOD1 F64A (**A**) or monomeric apoSOD1 (**B**) heated to 37°C (310 K). The solutions were further incubated for 4–5 h, where after a melting curve was obtained and compared to the thermal transition of each protein incubated without Zn^2+^. For F64A, the two experimental conditions resulted in indistinguishable melting curves, demonstrating very poor affinity of the protein for Zn^2+^. As expected, monomeric apoSOD1 incubated with Zn^2+^ results in a protein with significantly higher melting point, demonstrating high affinity for Zn^2+^.
(TIF)

Figure S4 **Addition of Zn^2+^ saves the cells from apoSOD1 induced cytotoxicity.** Cells were incubated with either 10 µM monomeric apoSOD1 [H46/48/120S] (**A**) or 1 µM monomeric apoSOD1 [H46/48/120S] (**B**) together with various metals. Protein and metals were added at the start of the experiment, and cells were subsequently incubated for 72 h. Cell viability was measured using the MTT assay. Proteins were added in triplicate and data are presented as mean and range as percentage of buffer control. The metal concentration in the culture medium was varied between 40 µM (red), 20 µM (purple), 10 µM (green), 5 µM (orange) and 2.5 µM (blue). Addition of Zn^2+^ in equimolar concentration saves the cells almost completely, indicating that Zn^2+^ detoxifies apoSOD1 by filling the empty Zn^2+^ site. None of the other metals tested, i.e. Fe^2+^, Fe^3+^. Ni^2+^, Mn^2+^ or Cu^2+^, have this effect. Addition of an excess concentration of metal to a lower concentration of apoSOD1 do not increase toxicity indicating that these metals do not interact with the protein in such a way that harmful free radicals can be formed or other toxic pathways commence.
(TIF)

Figure S5 **Monomeric apoSOD1 induces toxicity also under serum free conditions.** Cells were incubated with monomeric apoSOD1 in cell media without serum or under standard conditions, i.e. media supplemented with 0.5% serum. Cell viability was measured using the MTT assay. Proteins were added in duplicate and data are presented as mean and range as percentage of the buffer control. The effect of apoSOD1 on cells with or without serum is of a similar magnitude.
(TIFF)

Text S1 **Cell growth and treatment conditions for supporting figures and** **Figure 2** **.**
(DOC)
