# Supplementary figures and images for: Cytotoxicity of Superoxide Dismutase 1 in Cultured Cells Is Linked to Zn2+ Chelation
Source: PLoS One. 2012 Apr 25;7(4):e36104. doi: 10.1371/journal.pone.0036104 (PMC3338499; doi:10.1371/journal.pone.0036104)

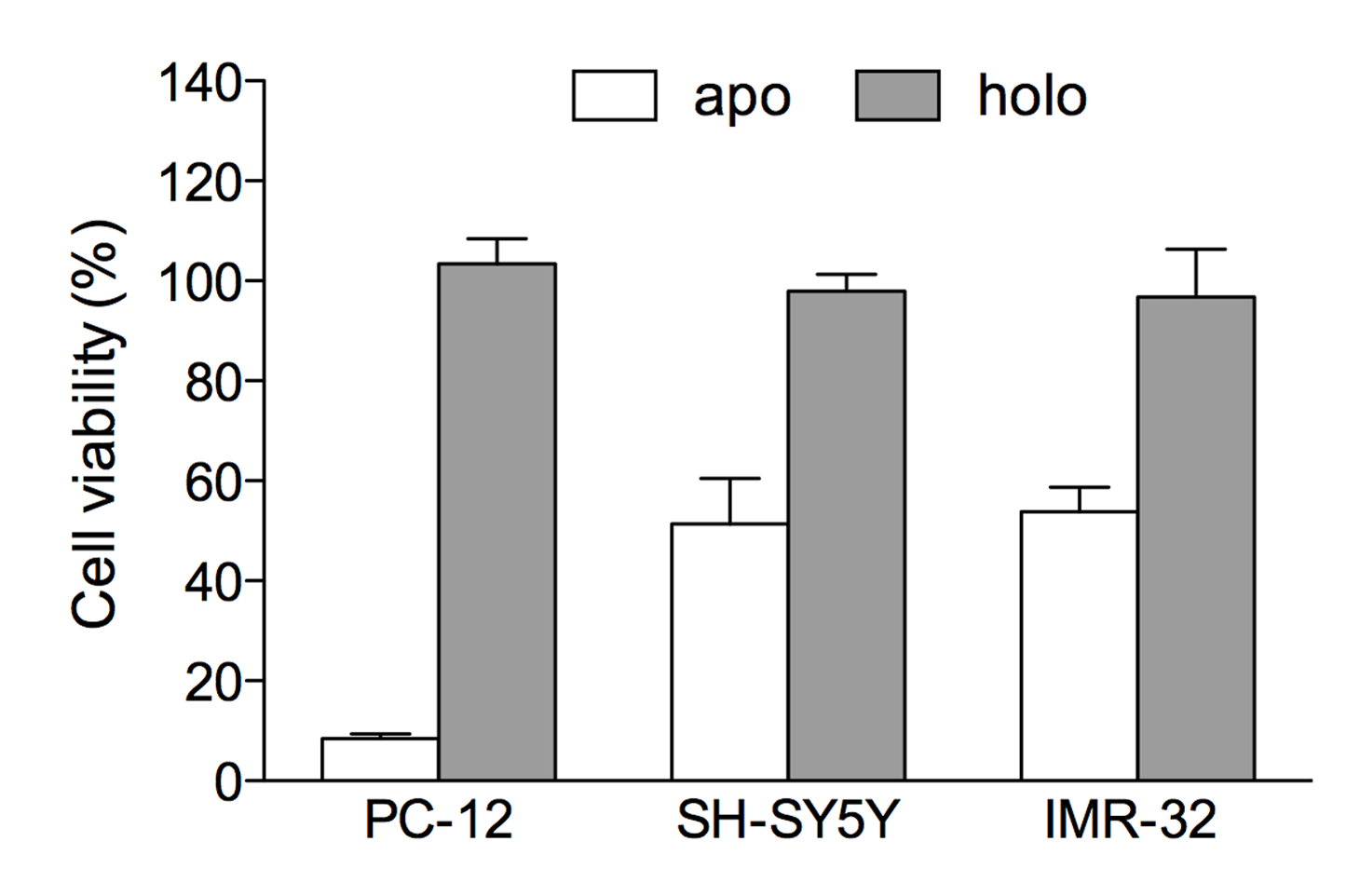

Supplement: Figure S1 — Dimeric apoSOD1 is cytotoxic to three different cell lines. Dimeric apoSOD1 was added to three different cell lines in a final concentration of 5 µM and incubated for 72 h. Cell viability was measured using the resazurin assay. Proteins were added in triplicate and data are presented as mean ± SD as percentage of the buffer control. ApoSOD1 (white bars) reduced cell viability in all three cell lines, whereas holoSOD1 (grey bars) was non-toxic. (TIFF) [file pone.0036104.s001.tiff]

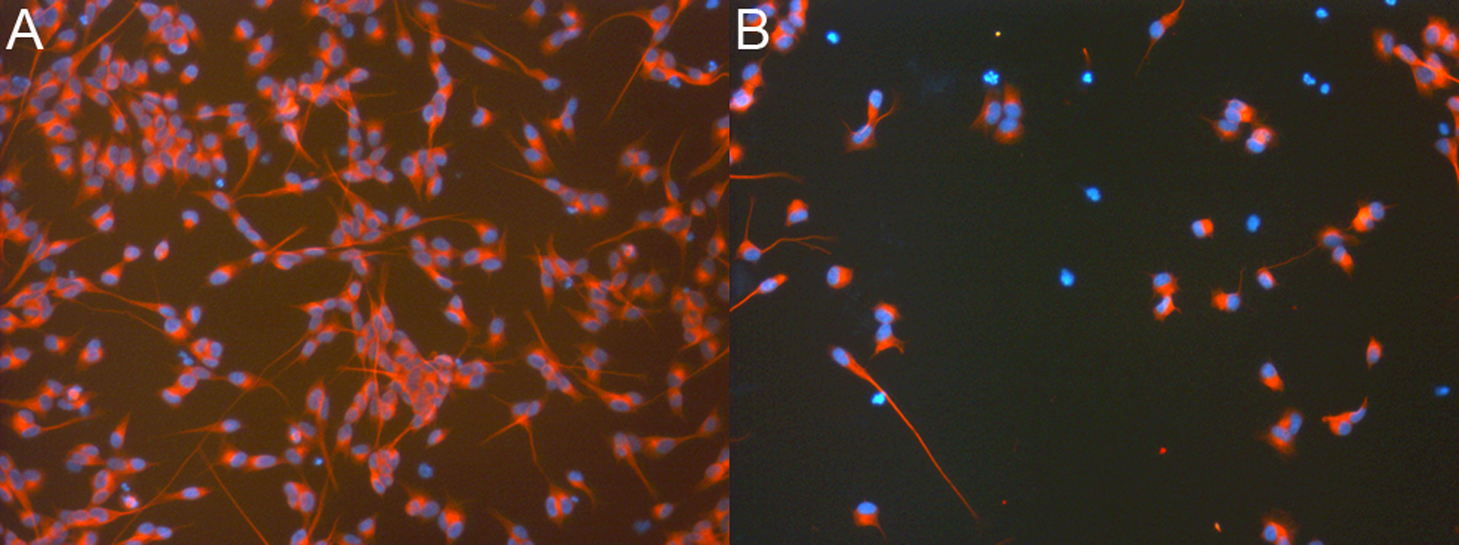

Supplement: Figure S2 — Fluorescence microscopy of cells exposed to apo and holoSOD1. Cells were visualized with immunocytochemistry, using DAPI for DNA staining (blue) and an anti-tubulin antibody for staining of the cytoskeleton (red). (A) Monomeric holoSOD1 (5 µM) does not cause any visible cell death after 72 h of incubation (200× magnification). (C) Few cells remain after exposure to 5 µM monomeric apoSOD1 for 72 h (200× magnification). (TIF) [file pone.0036104.s002.tif]

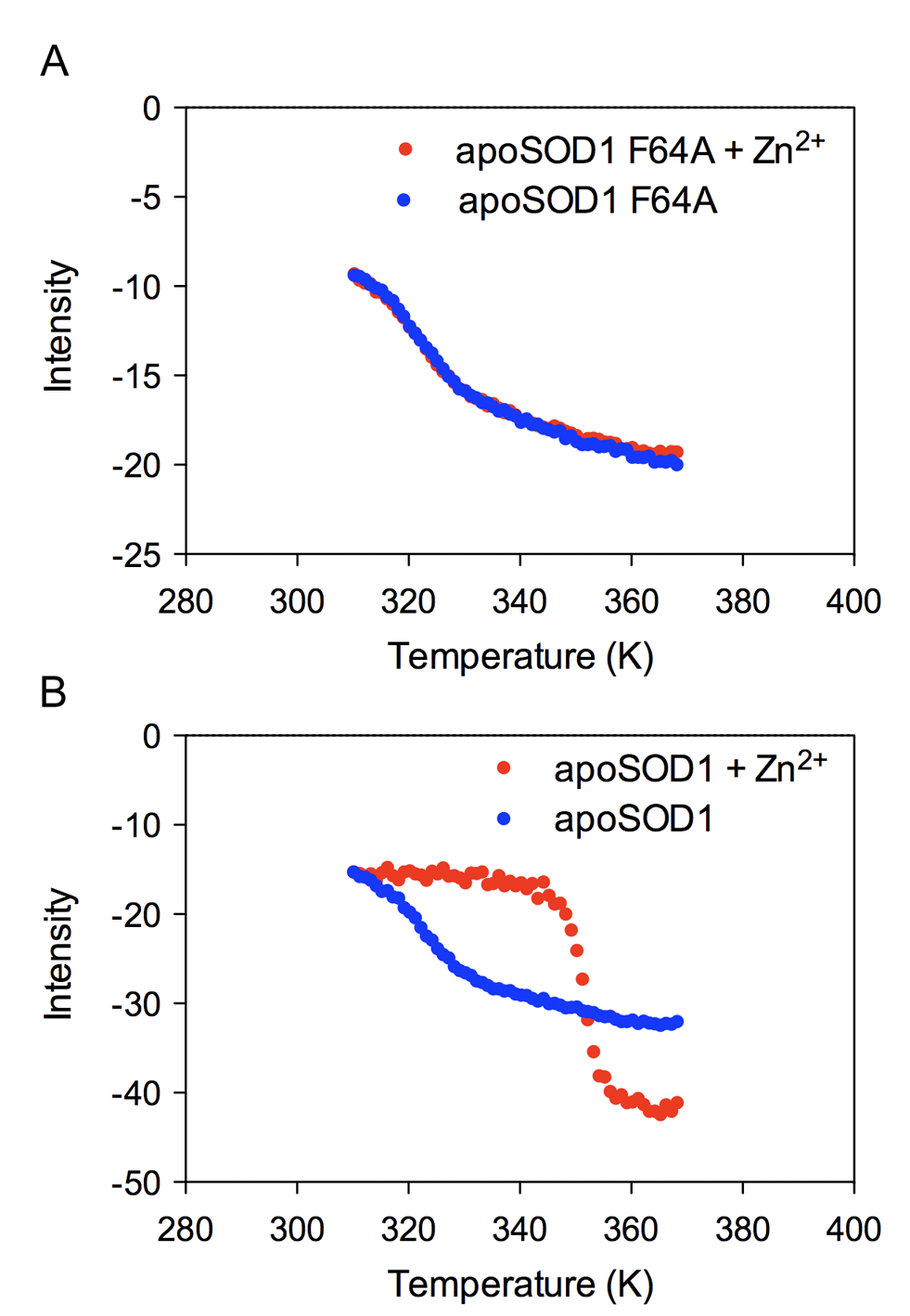

Supplement: Figure S3 — The thermal transition of apoSOD1 F64A is not affected by Zn2+. Zn2+ was added in 5 to 7 molar excess to a protein solution of monomeric apoSOD1 F64A (A) or monomeric apoSOD1 (B) heated to 37°C (310 K). The solutions were further incubated for 4–5 h, where after a melting curve was obtained and compared to the thermal transition of each protein incubated without Zn2+. For F64A, the two experimental conditions resulted in indistinguishable melting curves, demonstrating very poor affinity of the protein for Zn2+. As expected, monomeric apoSOD1 incubated with Zn2+ results in a protein with significantly higher melting point, demonstrating high affinity for Zn2+. (TIF) [file pone.0036104.s003.tif]

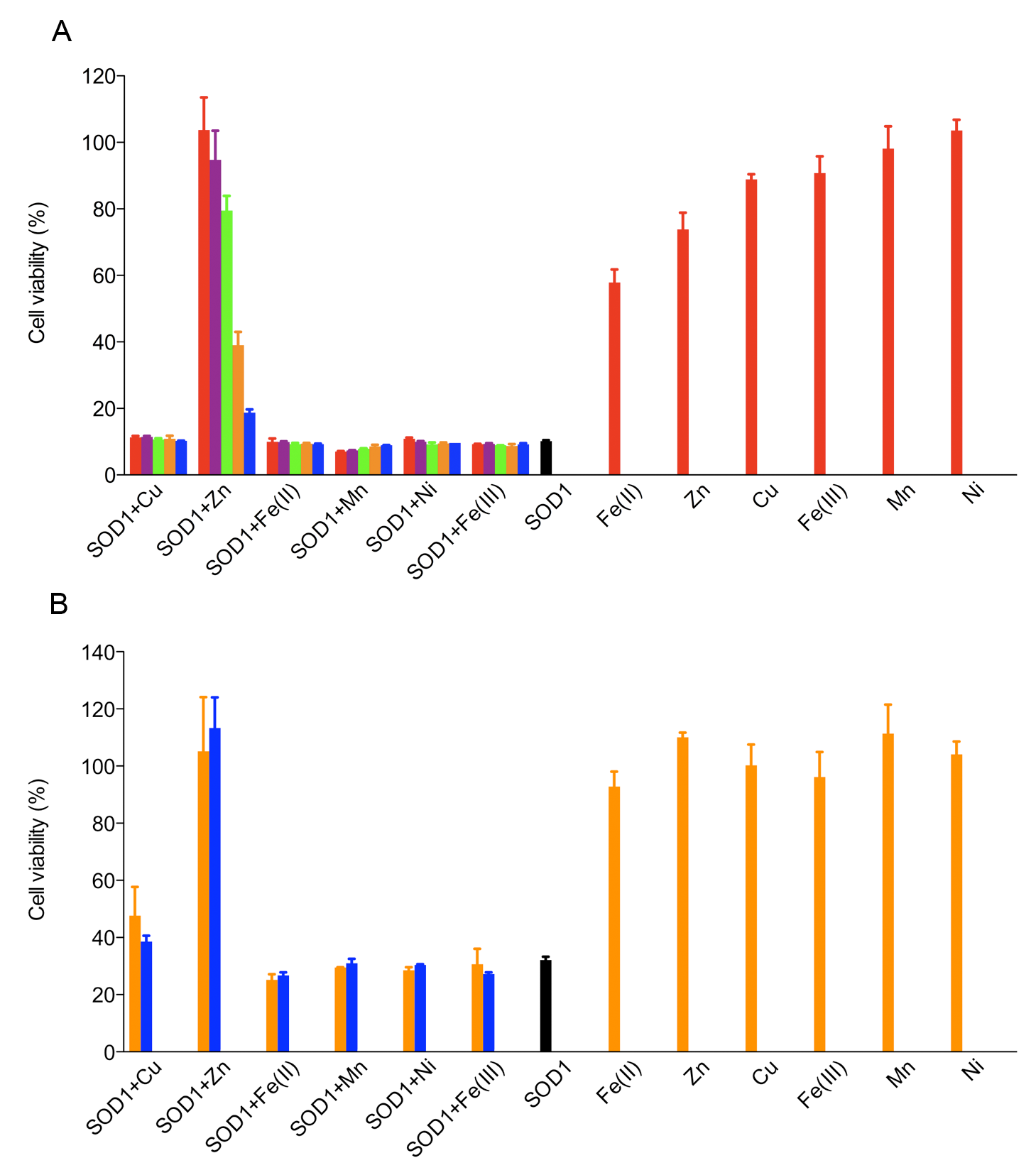

Supplement: Figure S4 — Addition of Zn2+ saves the cells from apoSOD1 induced cytotoxicity. Cells were incubated with either 10 µM monomeric apoSOD1 [H46/48/120S] (A) or 1 µM monomeric apoSOD1 [H46/48/120S] (B) together with various metals. Protein and metals were added at the start of the experiment, and cells were subsequently incubated for 72 h. Cell viability was measured using the MTT assay. Proteins were added in triplicate and data are presented as mean and range as percentage of buffer control. The metal concentration in the culture medium was varied between 40 µM (red), 20 µM (purple), 10 µM (green), 5 µM (orange) and 2.5 µM (blue). Addition of Zn2+ in equimolar concentration saves the cells almost completely, indicating that Zn2+ detoxifies apoSOD1 by filling the empty Zn2+ site. None of the other metals tested, i.e. Fe2+, Fe3+. Ni2+, Mn2+ or Cu2+, have this effect. Addition of an excess concentration of metal to a lower concentration of apoSOD1 do not increase toxicity indicating that these metals do not interact with the protein in such a way that harmful free radicals can be formed or other toxic pathways commence. (TIF) [file pone.0036104.s004.tif]

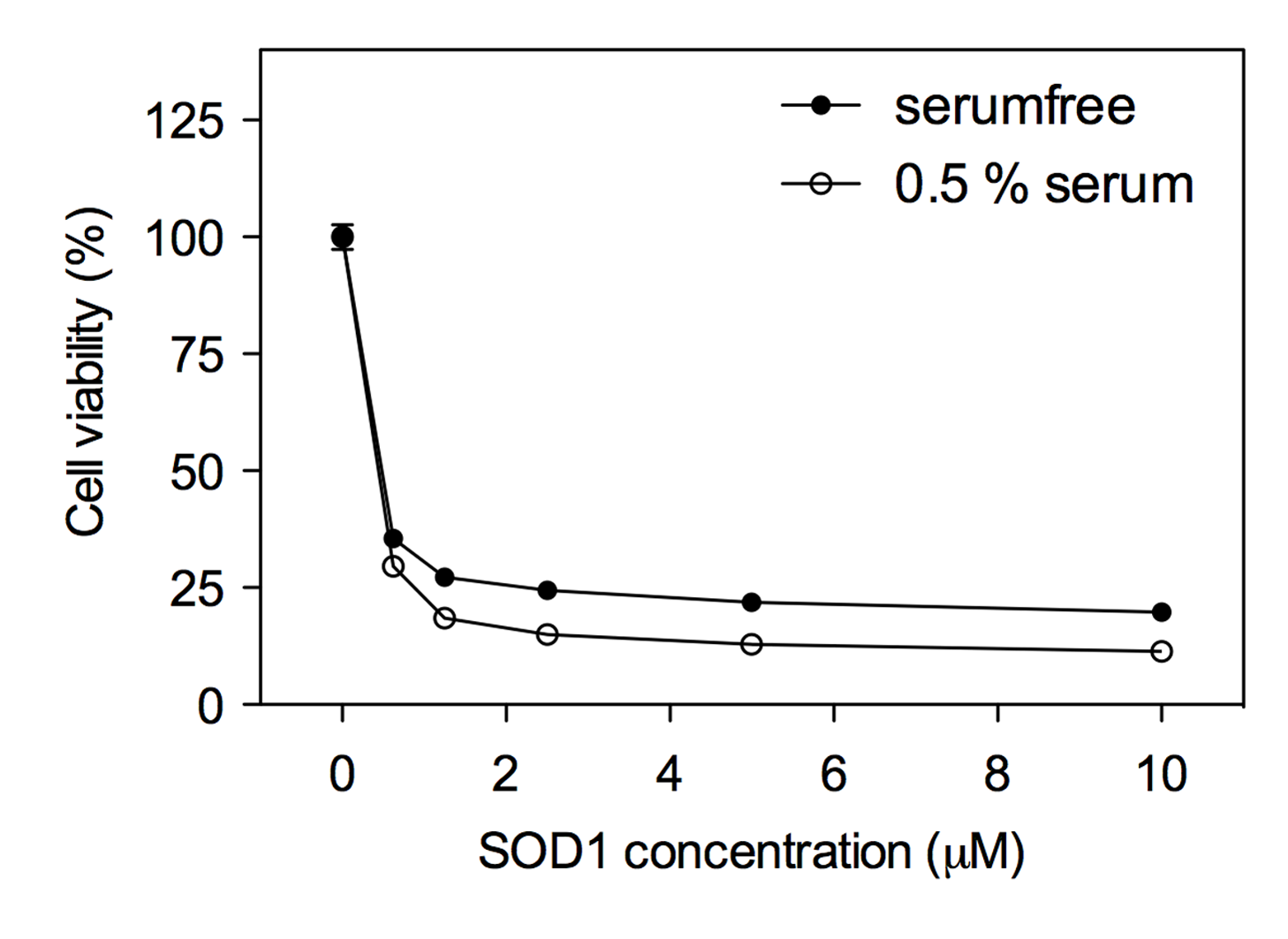

Supplement: Figure S5 — Monomeric apoSOD1 induces toxicity also under serum free conditions. Cells were incubated with monomeric apoSOD1 in cell media without serum or under standard conditions, i.e. media supplemented with 0.5% serum. Cell viability was measured using the MTT assay. Proteins were added in duplicate and data are presented as mean and range as percentage of the buffer control. The effect of apoSOD1 on cells with or without serum is of a similar magnitude. (TIFF) [file pone.0036104.s005.tiff]
