# Supplementary material for: Cytotoxicity of Superoxide Dismutase 1 in Cultured Cells Is Linked to Zn2+ Chelation
Source: PLoS One. 2012 Apr 25;7(4):e36104. doi: 10.1371/journal.pone.0036104 (PMC3338499; doi:10.1371/journal.pone.0036104)
Supplement: Text S1 — Cell growth and treatment conditions for supporting figures and Figure 2 . (DOC) [file pone.0036104.s006.doc]

Supporting information:

Cell growth and treatment conditions

Ann-Sofi Johansson *et al.*

**Growth conditions for experiments in Figure 2 and Figure S1.** Human neuroblastoma SH-SY5Y cells were cultured in Minimum Essential Medium (MEM) with GlutaMAX^TM^ I and Earle’s salt (Gibco), supplemented with 10% fetal bovine serum (FBS), 100 units/ml penicillin, 100 µg/ml streptomycin, and 1% Non-essential amino acids solution (NEAA) (Gibco, Invitrogen, Sweden). Human neuroblastoma IMR-32 cells (CCL-127) were cultured in MEM with GlutaMAX^TM^ I and Earle’s salt, supplemented with 10% FBS, 100 U/ml penicillin and 100 μg/ml streptomycin, 1 mM sodium pyruvate and 1% NEAA. PC12 cells from rat pheochromocytoma were cultured in RPMI 1640 (Gibco), supplemented with 10% horse serum, 5% fetal bovine serum and 50 units/ml penicillin, 50 µg/ml streptomycin. All cells were cultured at 37°C in a humidified atmosphere of 5% (v/v) CO_2_/air.

**Cell treatment conditions for experiment in Figure S1.** SH-SY5Y and IMR-32 cells were seeded in supplemented MEM at a density of 40 000 cells/cm^2^ (PC12 cells were seeded at a density of 2000 cells/cm^2^) in 96-well plates and incubated for 24 h prior to addition of purified apo or holo protein diluted in supplemented MEM without serum and further incubation for 72 h. All cell lines were detached using trypsin.

**Cell treatment conditions for serum-free experiment in Figure S3.**  SH-SY5Y were detached mechanically (to avoid traces of active trypsin) and seeded in serum free DMEM at a density of 57 000 cells/cm^2^ in 96-well plates and grown over night prior to addition of SOD1. Prior to treatment, serum free or DMEM supplemented with 1% serum was added resulting in totally serumfree conditions or a final serum concentration of 0.5%.
